# Central vascular ligation and mesentery based abdominal surgery

**DOI:** 10.1007/s12672-021-00419-4

**Published:** 2021-08-06

**Authors:** M. Franceschilli, D. Vinci, S. Di Carlo, B. Sensi, L. Siragusa, A. Guida, P. Rossi, V. Bellato, R. Caronna, S. Sibio

**Affiliations:** 1 Department of Surgical Sciences, Minimally Invasive Surgery Unit, University of Rome “Tor Vergata”, Rome, Italy; 2 Department of Surgery Pietro Valdoni Unit of Oncologic and Minimally Invasive Surgery, Rome, Italy; 3 Department of Surgical Science, Sapienza University of Rome, Rome, Italy

**Keywords:** Complete mesenteric excision, Central vascular ligation, Colorectal cancer, Gastric cancer, Pancreatic head cancer, Lymphadenectomy, Laparoscopy, Minimally invasive approach

## Abstract

In the nineteenth century the idea of a correct surgical approach in oncologic surgery moved towards a good lymphadenectomy. In colon cancer the segment is removed with adjacent mesentery, in gastric cancer or pancreatic cancer a good oncologic resection is obtained with adequate lymphadenectomy. Many guidelines propose a minimal lymph node count that the surgeon must obtain. Therefore, it is essential to understand the adequate extent of lymphadenectomy to be performed in cancer surgery. In this review of the current literature, the focus is on “central vascular ligation”, understood as radical lymphadenectomy in upper and lower gastrointestinal cancer, the evolution of this approach during the years and the improvement of laparoscopic techniques. For what concerns laparoscopic surgery, the main goal is to minimize post-operative trauma introducing the “less is more” concept whilst preserving attention for oncological outcomes. This review will demonstrate the importance of a scientifically based standardization of oncologic gastrointestinal surgery, especially in relation to the expansion of minimally invasive surgery and underlines the importance to further investigate through new randomized trials the role of extended lymphadenectomy in the new era of a multimodal approach, and most importantly, an era where minimally invasive techniques and the idea of “less is more” are becoming the standard thought for the surgical approach.

## Introduction

According to GLOBOCAN 2018 [1] cancer incidence is increasing because of ageing societies, commercial interests, and unhealthy lifestyles. Currently, one in five men and one in six women will be diagnosed with cancer, and one in eight men and one in ten women will die from their disease. Predictions suggest that by 2030, 30 million people will die from cancer each year.

Metastatic disease is the main cause of deaths from cancer; therefore, prevention of later arising metastasis has moved to the center of clinical attention. Prevention of metastasis combines early surgery (or radiotherapy in some cases) with systemic therapy, neoadjuvant or adjuvant. Systemic therapy mainly targets tumor cells that have detached from the primary lesion to lodge elsewhere, undetectable by clinical imaging and inaccessible to excision [2].

During the nineteenth century the idea of a correct surgical approach moved towards a good lymphadenectomy. In colon cancer the segment is removed with adjacent mesentery, in gastric cancer or even in pancreatic cancer a good oncologic resection is obtained with adequate lymphadenectomy.

The Halsted and the Fisher model represent the lymphatic spread biological models; in the former the lymphatic spread follows a well-defined temporal and anatomical path, from the primary tumor to nearby lymph nodes (LN), henceforth to intermediate nodes, subsequently to central nodes, and eventually to distant organs such as the liver or lung [2]; in the latter, the lymphatic, as well as hematogenous metastases, occur early and at random [3].

It seems essential to understand the adequate extent of lymphadenectomy to be done in cancer surgery and indeed many guidelines propose a minimal lymph node count (LNC) that the surgeon must obtain.

Notably, the literature often uses the terminology of Complete Mesocolic Excision (CME) and CME with central vascular ligation (CVL) interchangeably.

CME was defined as dissection between the right mesocolon and the retroperitoneum, following the embryological plane defined by the fusion fascia of Toldt and the fusion fascia of Fredet and high tie of ileocolic vessels, right colic vessels, and right branch of middle colic vessels [4, 5]. The CME procedure requires proximal vascular ligation of the feeding vessels but does not specify nor require dissection at the level of the root vessels [superior mesenteric vein and artery (SMV, SMA)] [6]. The original CME technique emphasized meticulous dissection between the mesocolon and retroperitoneum along the Toldt’s fascia and retrieval of the specimen as one unbreached mesocolon package. CME should be performed with techniques of central vascular ligation (CVL) to clear all locoregional lymph nodes. In addition, adequate proximal and distal margins should be obtained.

**Fig. 1:**
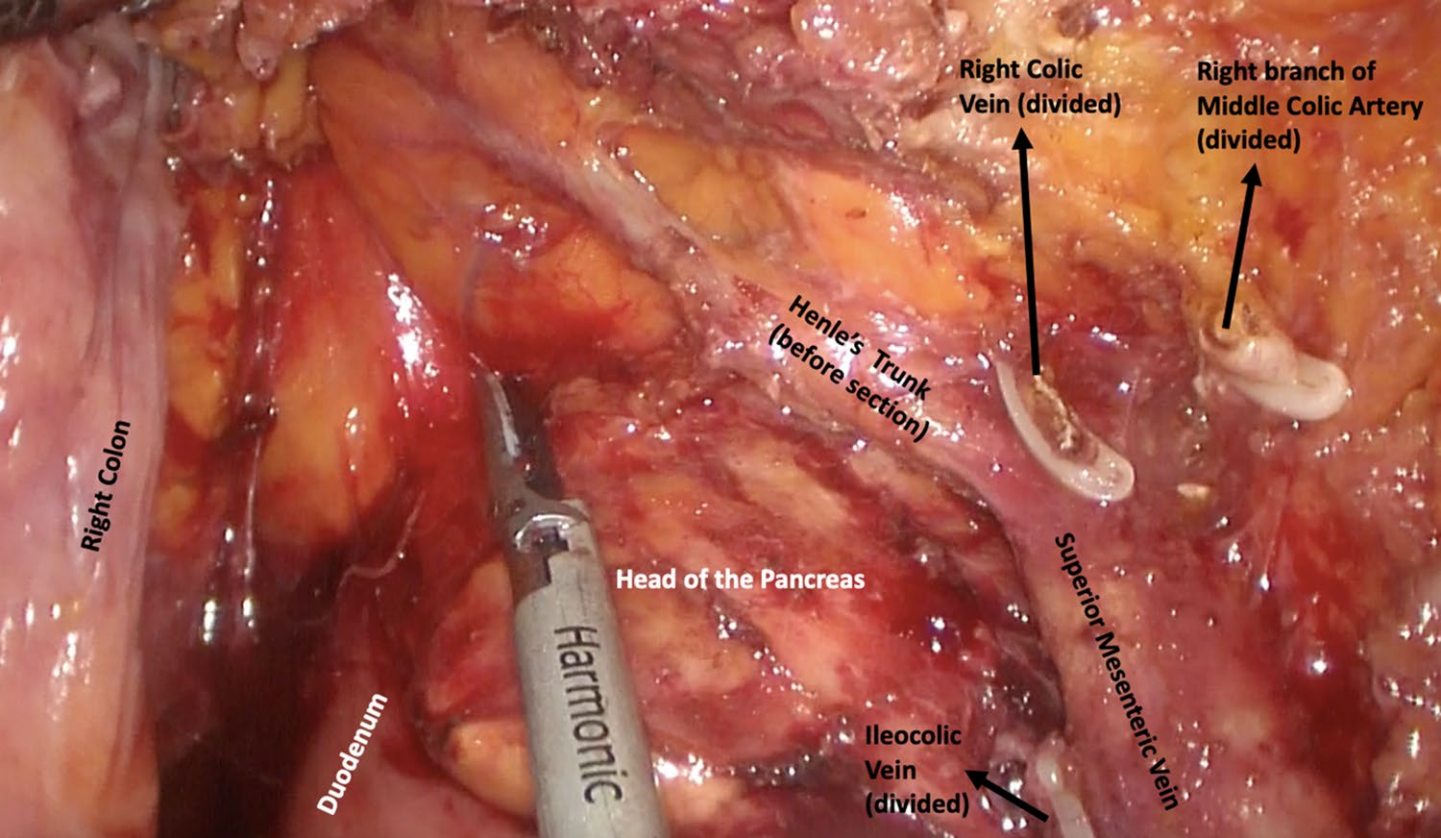
Complete Mesocolic Excision during right colectomy: lymphoadipose tissue covering the head of pancreas after section of the superior right colic vein (SRCV) at its confluence in the gastrocolic trunk of Henle (before dissection), and right branch of Middle colic artery

**Fig. 2:**
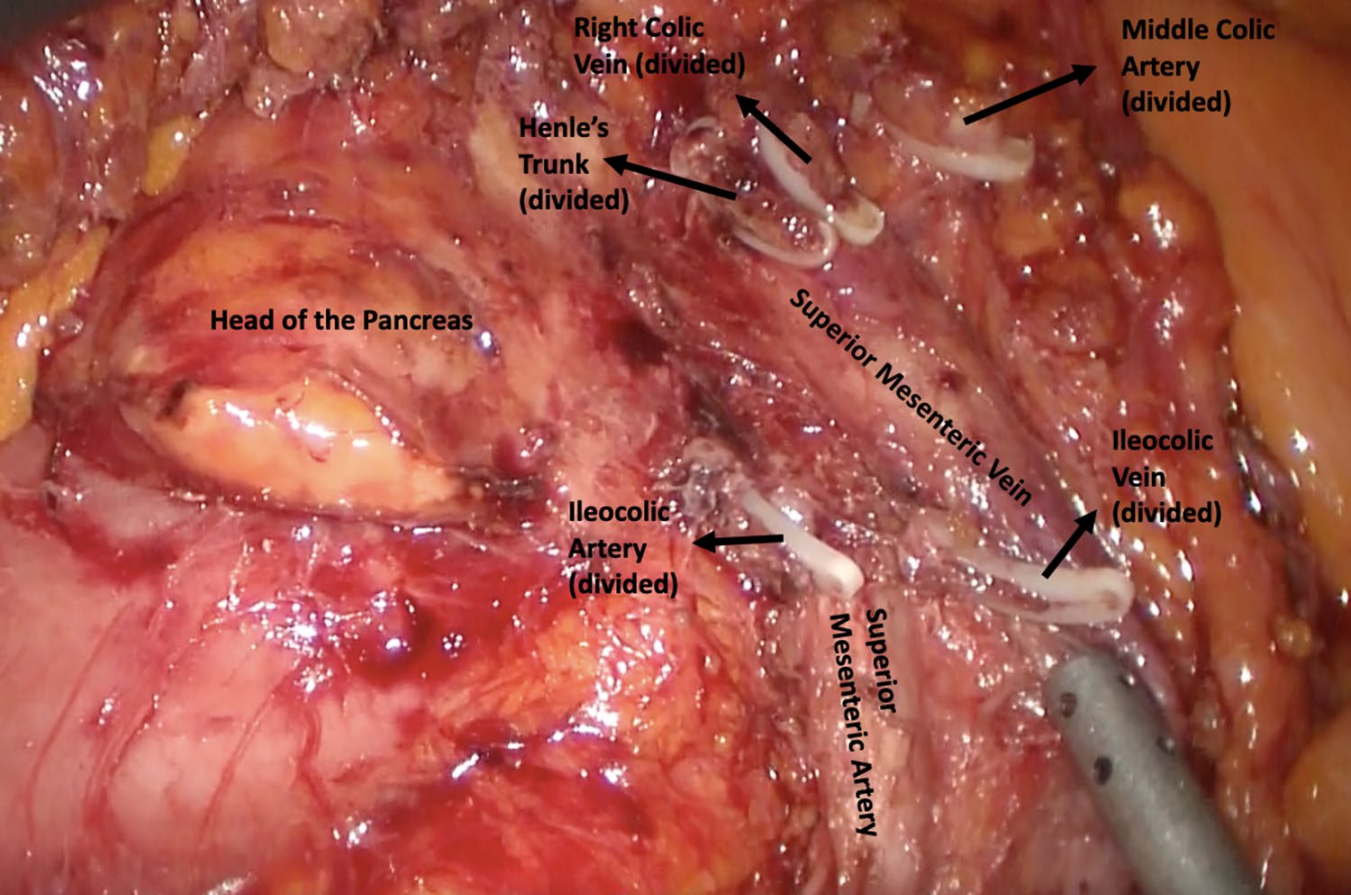
Complete Mesocolic Excision during right colectomy: lymphoadipose tissue covering the head of pancreas after section of the superior right colic vein (SRCV) at its confluence in the gastrocolic trunk of Henle (after dissection), and right branch of Middle colic artery

To be more specific, D1 lymph node resection represents transection of the mesenteric vessel just proximal to the marginal vessels; D2 resection is a more traditional resection of the main feeding vessels to a given colonic segment and lymphadenectomy that includes the origin of the feeding vessels [7]; D3 represents an extended lymphadenectomy that includes dissection of the lymphoadipose tissue covering the medial side of the SMV and dissection of the lymphoadipose tissue covering the head of pancreas after section of the superior right colic vein (SRCV) at its confluence in the gastrocolic trunk of Henle (GCTH) if necessary (Figs. 1, 2). The latter is a fundamental surgical landmark defined as the venous confluence of the following three veins: right gastroepiploic vein, anterosuperior pancreatic-duodenal vein, and SRCV [4].

Although there are some differences in the extent of surgery between CME and D3 lymphadenectomy, these two surgical techniques (if CME is properly performed) share common characteristics regarding oncologic outcomes. Moreover, it is author’s opinion that a true CME does not exist without CVL and extended dissection along the vascular plane offered by the anterior surface of the SMV.

Hohenberger et al. [8] firstly described CME in conjunction with (CVL) meaning that (CVL) includes individual proximal vascular ligation, with extended central lymph node dissection.

Furthermore, the introduction of minimally invasive surgery (MIS), that has revolutionized gastrointestinal surgery [9] introducing the concept of “less is more”, has introduced further issues in oncologic abdominal surgery.

Specific concerns on the oncological outcomes of procedures such as gastrectomy, pancreatectomies and rectal resections, are extensively debated, especially for what concern laparoscopic lymphadenectomy. The development of newly designed operative techniques and the introduction of better technological devices for laparoscopic and robotic surgery together with the undoubted improvement of surgical expertise in minimally invasive surgery, might overcome this problem [10–13].

## Materials and methods

Between 1 March 2021 and 31 March 2021, a review of the literature has been performed for articles published between 1990 and 2021. The following databases were searched: MEDLINE (PubMed), EMBASE (OvidSP), Ovid, and Cochrane Database. The following search terms were used: “Central Vascular Ligation”, “Mesenteric excision”, “Colorectal cancer”, “Gastric Cancer”, “Pancreatic Cancer”, “Oncologic outcome”, “Laparoscopic approach”, “Minimally invasive approach” and “Complete Mesenteric Excision”. Furthermore, cross searches were performed through references from considered articles. Two screeners independently performed the literature screening and review (DV and MF). Discrepancies were addressed by agreement with a third screener (SS).

## Results

All 712 articles have been analysed and those which met the eligibility criteria were reviewed. Data from a total of 73 different full articles was extracted. Duplicates, articles in languages other than English and articles not relevant to this review were excluded (Table 1). Articles included preclinical studies, retrospective analyses, case reports/case series, observational cohort studies, randomized clinical trials.

**Table 1:**
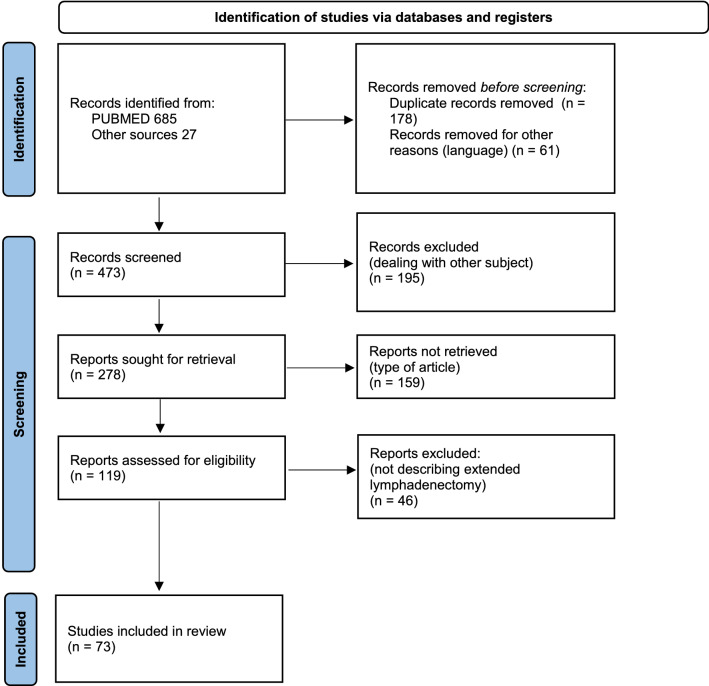
PRISMA 2020 flow diagram for new systematic reviews which included searches of databases and registers only

## Discussion

### Colorectal cancer

Colorectal cancer is the second cause of cancer-related death in Western countries and surgical resection is still the main treatment. It is supposed to be caused by a complex interaction between environmental carcinogens and genetic alterations, which ultimately results in the uncontrolled growth of transformed cells; the cancer tissue is infiltrated with various immune cells, which can either promote or inhibit CRC cell growth [14–18].

The primitive dorsal mesenterium is the embryological “envelope” made of a double-layered mesenchymal fibrofatty tissue, also route for cancerous diffusion from the primitive tumor. The complete excision of this “meso-structure” is thus crucial to enhance clearance of the surgical field, and to avoid local recurrence. Central vascular ligation (CVL) permits an extensive lymph node dissection, limiting regional recurrence and systemic dissemination, possibly providing improved survival in stage I-III colonic cancers [19].

The concept of total mesorectal excision (TME) championed by Heald [4] has shown that good quality surgery improves the outcome for rectal cancer. TME, removing the primary lymph-vascular drainage as an intact package together with the tumor and preserving the autonomic nerves required for the maintenance of urinary and sexual function, is nowadays accepted as the gold standard for rectal cancer surgery [20]. The dissection plane described by Heald, remains the single, most important prognostic factor for local recurrence. In fact, recent meta-analyses and reviews comparing TME plus lateral lymph node dissection (LLND) versus TME alone [21–24], could not find significant reduction of recurrence rates or improvement in survival; however, LLND is reported to require longer operation time (360 min versus 294.7 min, P = 0.02) and to be associated to increased complication rates (odds ratio = 1.48, 95% confidence interval 1.18–1.87, P < 0.001) such as urinary dysfunction [19].

In recent years, the concept of CME with dissection adhering to embryological planes and central vascular ligation (CVL) has also been adopted to colonic resection [5, 25]. While data exist describing an increased disease-free survival (DFS) in patients who have undergone right colectomy using CME [26, 27], little is known about the perioperative morbidity and mortality associated with CME/CVL [28]. Hohenberg et al. [8] proposed a nodal dissection even more extended than the standard D3 proposed by Japanese surgical societies, known as CVL.

Lymph node ratio is a well-known prognostic factor as associated with a better DFS in colorectal cancer and an extended lymphadenectomy with preservation of mesocolic plane demonstrated a relevant increase of node harvested and consequently a lower node ratio [29, 30]. Despite this encouraging result, the mechanism underlying how a lower node ratio impact in recurrence rate, DFS and OS, still remains unclear.

Extended lymphadenectomy requires a wider dissection around large vessels that makes the colorectal resection more demanding, especially in right colon resections. Ferri et al. [31] conducted a meta-analysis including a total of 3918 patients. The CME group with respect to conventional right-side colectomy showed a better five-year DFS and overall survival (OS) with an OR 1.88 (95% CI 1.02–3.45) and OR 2.77 (95% CI 1.33–5.74), respectively. In addition, the two groups had a similar incidence of mortality and morbidity. Moreover, in the CME group a higher mean number of lymph nodes has been obtained (MD 7.08 lymph nodes 95% CI 4.90–9.27). On other hand other studies did not demonstrated CME superiority so that CME still cannot be offered as the standard of care [32].

Due to this aspect, though the oncological result seems promising, patient selection still needs to be standardized as the greater surgical complexity could expose patients to a higher complication rate without a definite oncological benefit, especially for early-stage cancer. Indeed, an Italian randomized trial (NCT04871399) is now comparing in safety and oncological outcome of right hemicolectomy with complete mesocolon excision versus standard right hemicolectomy.

Another aspect yet to be evaluated is preoperative anatomy. Since dissection along major colorectal vessels is mandatory for a proper complete mesocolon excision, systematic preoperative CT-scan evaluation of vascular anatomy seems reasonable in order to know anatomical variations, avoid vascular complications and obtain a more radical resection [33, 34]. Even in this case, while cadaveric study and model evaluating colorectal vascular anatomy already exists, still no strong evidence is able to verify this hypothesis, despite an ongoing Norvegian randomized trial (NCT01351714) is evaluating oncological radicality in patients studied preoperatively with CT-scan with contrast.

Kanemitsu et al. [35] examined 370 consecutive patients who had right colectomy with D3 lymphadenectomy for right colon cancer; 3% of patients had N3 nodal involvement (patients with T3-T4 tumors) and 13.2% had N2 nodal involvement. The 5-year DFS was 36.4% for the patient with N3 nodal involvement versus 83.5% for N2 nodal involvement, suggesting that patients with proximal nodal metastasis exhibit a different tumor biology than patients with more intermediate-level nodal metastasis. According to Nagasaki et al. [36] lymph nodes are a key element of TNM staging system and are considered a significant factor for predicting DFS and OS in patients with colorectal cancer without distant metastasis. Regional lymph node metastasis is believed an essential step in tumor cell dissemination in colorectal cancer. Furthermore, in colorectal cancer surgery, intraperitoneal free cancer cells (IFCC) presence is not routinely investigated and their prognostic meaning is still unclear; yet when peritoneal washing results are positive for the presence of IFCC a worse outcome can be expected [37–39].

DFS is also related to the histotype; in a recent study it has been demonstrated that patients with pT3 mucinous and signet ring cell tumors resected after emergency presentation have poorest outcomes and survival. Furthermore, the debate of whether emergency colon surgery is associated with worse oncological outcome is still ongoing and finding a “bridge to surgery” strategy (if possible) might provide better oncologic outcomes in T3 patients [40].

Moreover, while Kotake et al. [41] demonstrated no difference in OS of patients who had T2 colon cancers treated with D2 or D3 resection, Slanetz et al. [42] showed that the level of mesenteric resection influenced outcomes only for the patients who had moderate or well-differentiated cancer with intermediate-level nodal involvement. Patients with more than four positive lymph nodes or poorly differentiated tumors had poor survival regardless of the extension of lymphadenectomy. These studies had limitations such as outdated staging methods, lack of modern chemotherapy, and no audit of the pathology specimen.

It is known that the 2019 guidelines of the Japanese Society for Cancer of the Colon and Rectum (JSCCR) recommend D3 lymph node dissection for clinical stage II/III colorectal cancer [43]. However, when performing a left hemicolectomy, it is still unclear whether D3 lymph node dissection with preservation of the left colic artery (LCA) is different in terms of clinical outcomes, compared to D3 without LCA preservation. The randomized controlled trial (RCT) JCOG0404 conducted by the Colorectal Cancer Study Group of the Japan Clinical Oncology Group (JCOG) [39] enrolled more than 1000 patients. The advantages in D3 without LCA preservation have been identified in the prevention of the micrometastatic cells spillage through the en-bloc lymph node dissection of the root of the inferior mesenteric artery (IMA); disadvantages include a higher possibility of anastomotic leakage and the sacrifice of autonomic nerves around the IMA; no significant differences in terms of operation time and blood loss have been found. Despite a higher complication incidence, D3 with LCA preservation was associated with a higher OS [44–48].

Furthermore, left hemicolectomies can be approached both through an open and laparoscopic approach; no matter the privileged technique, two critical steps must be taken care of: a well-perfused bowel and a tension-free anastomosis [49]. The correct management of major vessels guarantees a proper perfusion while the mobilization of proximal colon gives the opportunity to achieve a tension-free anastomosis; the proximal colon cannot be always fully mobilized and, in some cases, the proximal colon will not go easily to the pelvis down to the left retroperitoneum and to the left of the ligament of Treitz; in these cases, the retroileal routing technique can be considered as an alternative technique in order to gain mobility of the colon when the transverse colon does not seem to assure a tension-free anastomosis when coming down to the rectum; this is a challenging technique when approached laparoscopically, but the hand-assisted laparoscopic technique (HAL) could provide a maneuverability and tactile feedback that could guarantee a safe performance of this surgical technique [50].

Finally, a promising prospective is the use of indocyanine green used during laparoscopy or robotic colorectal procedures for lymphatic mapping that may help to perform a correct CME. Small series were published showing its safety and feasibility, but the actual utility deserves further investigations [51].

### Gastric cancer

Gastric cancer is one of the main causes of death for cancer in the world [52]. Radical gastrectomy with regional lymphadenectomy before or after chemotherapy is the gold standard in the treatment of invasive cancers [53].

Lymph nodes involvement and local spread is one of the main features of gastric cancer and nodal invasion is mainly influenced by tumor depth [54]. Nodal spread gradually takes place radiating from the primary site [55] and nodal involvement is one of the most important prognostic factors.

**Fig. 3:**
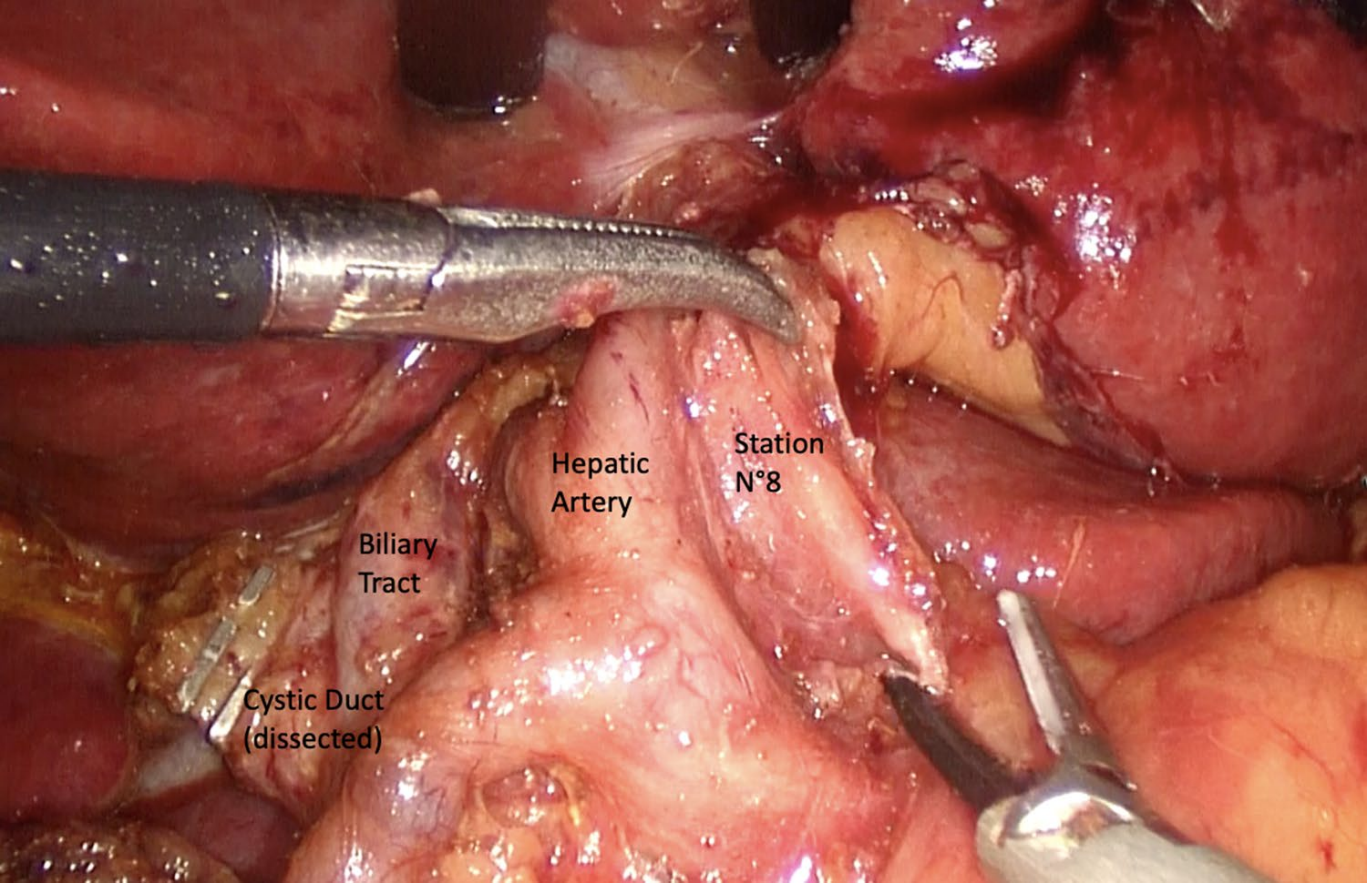
Lymphadenectomy D2 during Laparoscopic Gastrectomy

**Fig. 4:**
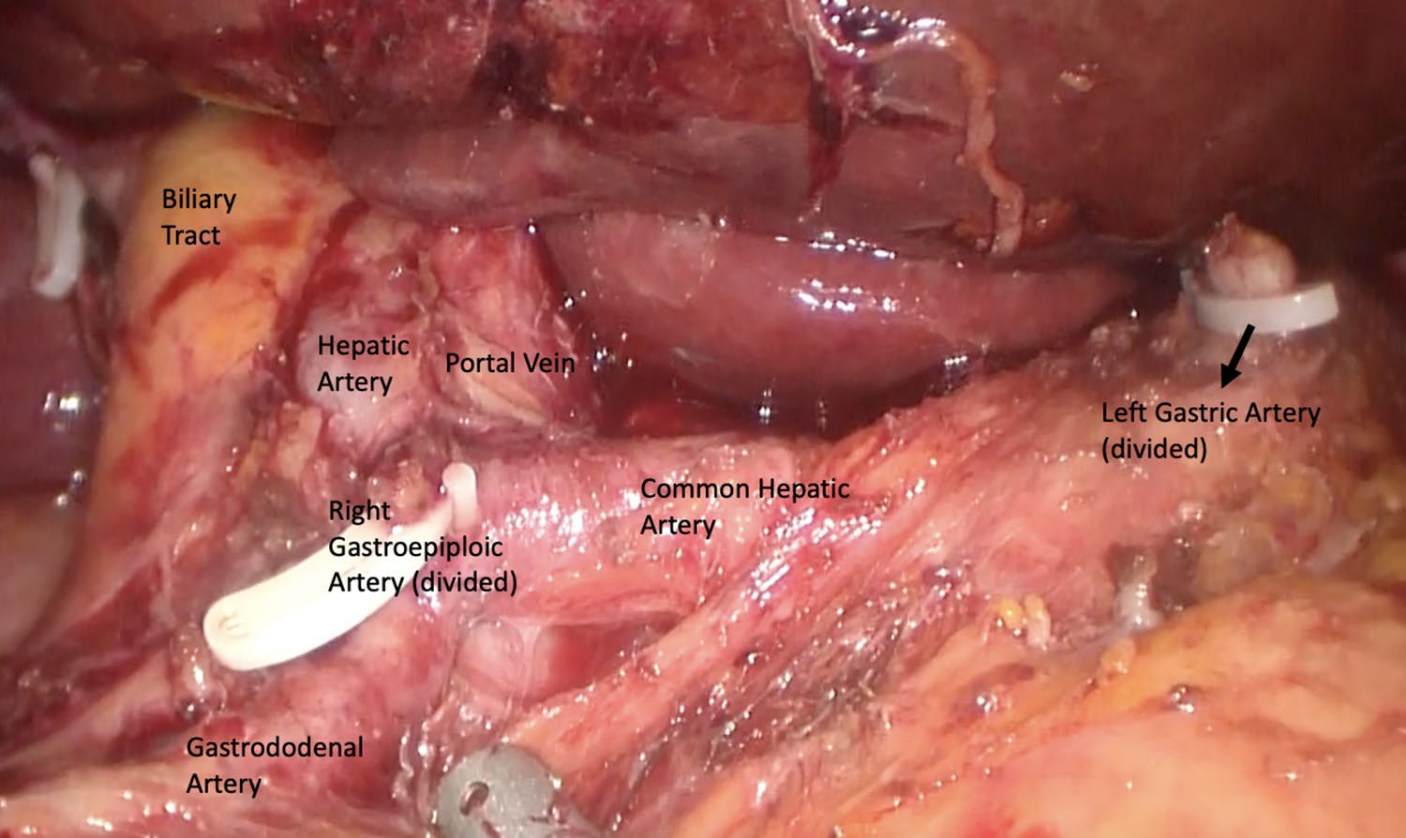
Lymphadenectomy D2 during Laparoscopic Gastrectomy (Hepatic Hilum dissection)

A D1 dissection aims to clear only the lymph nodes with the highest risk of involvement and thus all perigastric and left gastric artery lymph nodes are removed. In a D2 dissection, all D1 lymph nodes and lymph nodes along the main vascular axis of the stomach (hepatic and splenic as well as those along the coeliac axis) are removed (Figs. 3, 4). In addition, a D3 dissection has been described, which includes all D1 and D2 lymph node stations, supplemented with abdominal paraaortic and hepatoduodenal lymph nodes. East Asian observational studies as well as a randomized trial from Taiwan have convincingly shown a better survival with D2 gastrectomy than D1 gastrectomy [52]. However, the extent of lymphadenectomy has been debated for years especially in Europe and North America. The 15 year-follow up of the famous Dutch study [54] has demonstrated that loco-regional recurrence rate is significantly lower in patients treated with D2 lymphadenectomy versus patients who underwent D1 dissection. D3 lymph node dissection is not widely accepted. The study by Wu et al. [56] confirmed the higher risk of complications after extensive dissections and suggested that extensive lymph node dissection should be performed only by surgeons with extensive experience in the technique; post-operative hospital stay was longer for D3 patients [mean (SD) 19.6 (13.9) (range 10–98) d vs 15.0 (4.0) (range 10–30) d, P = 0.001]; morbidity rate was also higher (17.1% vs 7.3%, P = 0.012), mainly due to a high incidence of abdominal abscess (8.1% vs 0%, P = 0.003) and patients undergoing distal spleno-pancreatectomy had an even higher morbidity rate (35.7% vs 10.6%, P = 0.017). However, in both groups there was no operative mortality.

The Japanese JCOG 9501 trial concluded that routine addition of para-aortic dissection to D2 lymphadenectomy does not affect the rate and the pattern of recurrences in advanced gastric cancer [57]. The controlled randomized trial by Sasako et al. [58] comparing D2 lymphadenectomy alone with D2 lymphadenectomy plus para-aortic nodal dissection (PAND) concluded that treatment with D2 lymphadenectomy plus PAND does not improve the survival rate in curable gastric cancer. Nevertheless, D2 + PAND is feasible after neo-adjuvant chemotherapy [59] and the D3 dissection could be useful in subgroups of patients, as suggested by the rather high 5-year survival (18.2%) reported in the Japanese trial [60] in patients with pathologically positive para-aortic nodes after a prophylactic PAN dissection.

In Europe, Roviello et al. [61] demonstrated a potential benefit of super-extended lymphadenectomy in patients with advanced gastric cancer without serosal involvement. Following this lead, De Manzoni et al. [62] evaluated, through the largest series undergoing D3 lymphadenectomy in the Western world, the impact of super-extended D3 lymphadenectomy on overall and specific recurrences in a series of advanced gastric cancer patients; D3 lymphadenectomy reverses the negative impact of diffuse histotype on relapses, especially on locoregional recurrences. As a consequence, D3 could be considered a valid therapeutic option in histotype-oriented tailored treatment of AGC.

### Cancer of the head of the pancreas

The incidence of pancreatic cancer is increasing with 548.782 new cases predicted by 2030 [63]; its aggressive biological behavior causes a 76.7% recurrence rate at 25.3 months for resected patients with a median recurrence-free survival of only 11.7 months. It still represents one of the most lethal human malignancies with the 5-year survival to be as low as 5% [64]. Pancreatectomy is suitable for 15–20% of patients at initial diagnosis because most of them are diagnosed when locally advanced. Vascular involvement by the tumor assessed through radiological imaging gives us the opportunity to classify localized pancreatic adenocarcinoma (PA) in three categories: potentially resectable (PR) when there is no vascular involvement, borderline resectable (BR) with minor/moderate vascular involvement, and locally advanced (LA) in case unreconstructable venous occlusion or significant arterial involvement is present [65].

**Fig. 5:**
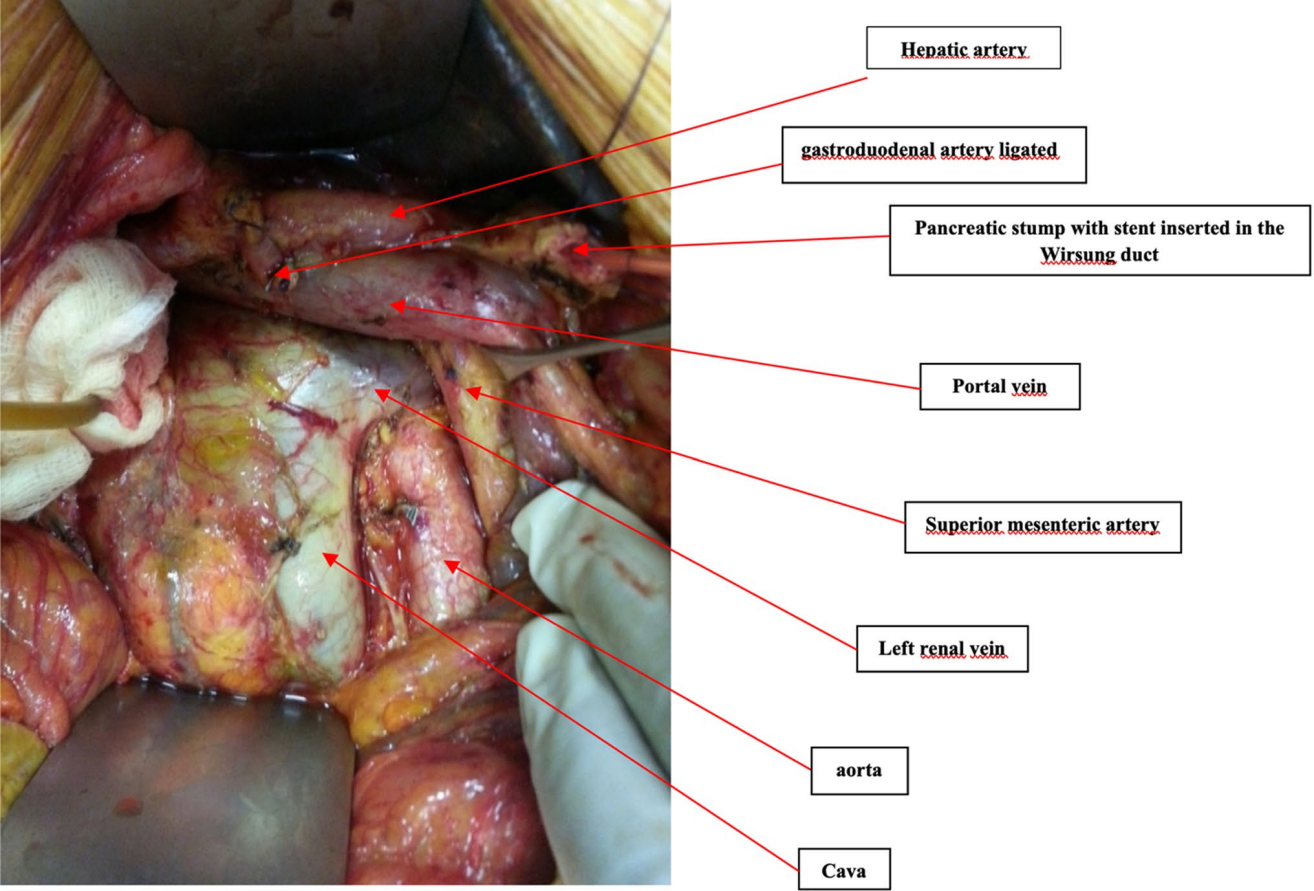
Complete mesopancreas excision during pancreatoduodenectomy

The pancreas is covered dorsally by a perineural layer, the mesopancreas, a well-vascularized structure extending from the posterior surface of the pancreatic head to behind the superior mesenteric vein and artery (SMV and SMA). The course of lymphogenic structures along the neuronal plexus posteriorly to the pancreas may have a key role in metastatic spread. Perineural tumor invasion has been detected in up to 77% of resection specimens from patients with carcinoma of the head of the pancreas [66]. Complete mesopancreas resection (CMR) and proximal jejunum excision have been advocated for R0 resection to achieve curability of a pancreatic head tumor, with direct or indirect invasion to major vessels or regional lymph node metastasis [67] (Fig. 5).

Lymph node metastases are found in 20–77% of resection specimens from patients with carcinoma of the head of the pancreas. However, the extent of lymphadenectomy does not exert a significant influence on the prognosis [68]. Four trials have been carried out in the last years [68–71] to support literature finding based only on several retrospective reports and few prospective randomized trials.

Pedrazzoli et al. [69] conducted a prospective, randomized, multicenter study to determine whether the performance of an extended lymphadenectomy and retroperitoneal soft-tissue clearance in association with a pancreatoduodenal resection improves the long-term survival of patients with a potentially curable adenocarcinoma of the head of the pancreas; an average of 13 lymph nodes were retrieved in the standard group, compared to 20 lymph nodes in the extended group. Statistically significant differences in the long-term survival of patients undergoing a standard or an extended lymphadenectomy has not been found. Henne-Bruns et al. [70] reported a prospective nonrandomized study in patients with pancreatic adenocarcinoma; on average, 14 lymph nodes were harvested in their standard procedure and 24 lymph nodes in their more extensive procedure. No survival benefit has been found for the more extensive procedure, with a median survival of only approximately 12 months in each group. The survival analysis conducted in the third trial, by Seiler et al. [71], revealed no differences in 1-year or 2-year survival rates or median survival when comparing the 33 patients undergoing classic pancreaticoduodenectomy (which includes a distal gastrectomy) to the 28 patients treated via pylorus-preserving pancreaticoduodenectomy. Yeo et al. [72], on the wave of the previously cited studies, conducted a prospective, randomized single-institution trial indicating that the addition of a distal gastrectomy (attended by harvesting of the respective perigastric lymph nodes) and retroperitoneal lymphadenectomy to pylorus-preserving pancreaticoduodenectomy provides no survival benefit.

The most recent literature finding about the role of node dissection in pancreatic tumor resection by Dillhoff et al. [73] stated that there are no data to support the empiric use of extended lymphadenectomy as a part of pancreaticoduodenectomy for patients with pancreatic adenocarcinoma.

### Laparoscopy and central vascular ligation: is it feasible?

The role of extended lymphadenectomy comes into an era where minimally invasive techniques and the “idea of less is more” is becoming the standard thought for the surgical approach. Gastrointestinal surgery has been revolutionized by the advent of laparoscopic and robotic surgery, reducing both post-operative trauma and the use of analgesics, and achieving a faster recovery.

To better investigate the issue of laparoscopic safety in general, several randomized trials were designed and carried out. Initial results with long-term follow-up showed no difference in survival and local recurrence rates when comparing laparoscopic to open approaches. The Laparoscopic approach showed some advantages over open surgery. The COST trial [74], COLOR I and II trials [75, 76], CLASICC trial [77, 78] and COREAN [79] demonstrated non-inferior outcomes to open surgery. Short-term benefits of the laparoscopic approach were also confirmed in a Cochrane Review: decreased blood loss, quicker oral intake, decreased narcotic use, and lower rates of surgical site infections [80]. Furthermore, Arezzo et al. [81] in a meta-analysis including 4539 patients found a mortality reduction (2.4% vs 1.0% P = 0.048) and decreased morbidity (35.4 vs. 31.8%, P < 0.001) in favor of the laparoscopic group.

In Negoi’s systematic review [82] no differences in local and distant recurrence rate, three- and five-year OS and DFS rates between Laparoscopic CME and Open CME groups were found.

Many specialized units performing laparoscopic CME/CVL have reported comparable quality of surgical specimens compared to open surgery [83, 84]. In one of the few RCTs, Yamamoto et al. compared laparoscopic and open D3 colonic resections and demonstrated lower morbidity rates in the laparoscopic group with the usual benefits of minimally invasive surgery [85].

A systematic review and network metanalysis conducted by Simillis et al. [86] took into consideration 29 randomized controlled trials accounting for 6237 participants and comparing: open versus laparoscopic versus robotic versus transanal mesorectal excision. Perioperative morbidity and long-term survival of the three techniques are perfectly comparable; an improved postoperative recovery could be observed in the laparoscopic and robotic approaches, and a better oncological margin resection characterized the open and transanal approaches; thus, concluding that the appropriate technique should be chosen depending on the surgeon expertise and on the patients’ expected benefits.

In recent years, several surgeons have started applying laparoscopic assisted gastrectomy (LAG) for gastric cancer [87–89]. A Phase III multicenter, prospective, randomized trial (KLASS Trial) by Kim et al. [90] evaluated morbidity, mortality and oncological safety of laparoscopic gastrectomy versus open gastrectomy for gastric cancer and showed no difference among the two approaches in the short-term outcomes giving hopes for no difference in the long-term. In the same way, Katai et al. [91] conducted a multicenter phase II trial (JCOG 0703) to evaluate the safety of laparoscopy-assisted distal gastrectomy (LADG) with nodal dissection for clinical stage I gastric cancer patients confirming the safety of laparoscopy-assisted distal gastrectomy (LADG) performed by credentialed surgeons in terms of the incidence of anastomotic leakage or pancreatic fistula formation. In a large scale of case—Control and case—Matched Korean Multicenter Study [92], at a median follow-up of 70.8 months, the OS, disease-specific survival, and recurrence-free survival were not statistically different except for patients with stage IA disease treated with laparoscopic surgery, who showed an increased OS (laparoscopic group; 95.3%, open group: 90.3%; P < 0.001) probably attributable to selection bias. Since short-term results and complication rate of laparoscopic gastrectomy with D2 lymph nodes dissection for advanced gastric cancer are still controversial, Lee et al. [93], in their center experience, concluded that laparoscopic assisted gastrectomy (LAG) with D2 lymphadenectomy is a feasible procedure for AGC, and the follow-up results demonstrated that it produced satisfactory surgical and oncological results, especially, for T2a and T2b. Despite the reverence held by many surgeons regarding the pancreas, within a few years after the introduction of laparoscopic cholecystectomy, laparoscopic surgery of the pancreas had been attempted [94]. LDP is increasingly applied to patients with high BMI, history of previous abdominal surgery, presence of comorbidities and large tumors. LDP has become the operation of choice for most lesions involving the distal pancreas [95]. Recent studies show that LDP have improved perioperative recovery and equivalent oncologic outcomes [96–99]. Studies on MIPD demonstrate that it is safe in terms of intra-operative outcomes, post-operative recovery and early oncologic outcomes. Laparoscopic enucleation has become the operation of choice for small benign tumors that are away from the main pancreatic duct, especially insulinomas [100]. Moreover, the advantages of the robotic approach such as a reduction of tremor, ergonomic movements, improved instruments and 3D view have given hint to a series of studies showing that robotic distal pancreatectomy is safe as the open and laparoscopic approach [101]. As stated by Ielpo et al. both robotic and laparoscopic distal pancreatectomies have similar peri-operative outcomes; the robotic approach is characterized by a lower conversion rate and hospital stay but a higher price; but the definition of cost is difficult to be given when factors such as quality of life, sexual function, sexual disorders, return to daily activities are taken into account; in addition, when compared to the laparoscopic approach, the robotic approach features easier instrument control and a more ergonomic position for the surgeon, which may be of particular importance when dealing with pancreatic surgery [102].

Central vascular ligation represents a universally accepted concept in colonic, rectal or gastric oncology, although its superiority regarding oncological outcomes is challenged by an increased rate of postoperative complications [98]. From anatomical, surgical, oncological and pathological perspectives we face different situations when approaching gastric, pancreatic, colonic or rectal cancers [103, 104].

Nowadays, TME is accepted as the gold standard in rectal cancer and tumor deposits as well as nodal involvement may be confined within the mesorectal fascia. Complete excision of the mesorectum should be performed en-bloc with the rectum by dissecting along the rectal fascia in the plane that separates this from the parietal pelvic fascia (the holy plane), thereby maintaining the integrity of the rectal fascia and mesorectal contents and sparing autonomic pelvic nerves and plexuses [105]. Furthermore, in recent years, the concept of CME with dissection adhering to embryological planes and central vascular ligation (CVL) has also been adopted to colonic resection [106, 107]. This will certainly affect the current value of current prognostic markers [108–113] that are essential for a modern precision oncology [114–121]

The “complete mesopancreas excision”, instead, is not similar to “complete mesocolon” or “total mesorectal” excision [122]. Extended lymphadenectomy has no survival benefit for patients with pancreatic cancer. Therefore, we suggest that standard lymphadenectomy should be favored over extended lymphadenectomy for pancreatic cancer. Routine extended lymphadenectomy has increased morbidity, operating room time, and length of stay, without evidence in improvement in OS; laparoscopic dissection of areas interested in CME with D3 extended lymphadenectomy could be associated with intra-operative bleeding, which may result fatal for the patient [123].

## Conclusion

Our intention is to increase the awareness that in an era characterized by progresses in surgical technique, it is important not to forget to locate the idea of extended lymphadenectomy and central vascular ligation in the context of a multimodal approach where neoadjuvant chemotherapy is increasing its role. Hence, our suggestion to further investigate through new randomized trials the role of extended lymphadenectomy in the new era of a multimodal approach, and most importantly, an era where minimally invasive techniques and the idea of “less is more” is becoming the standard thought for the surgical approach.

The achievement of a reliable laparoscopic lymphadenectomy in terms of oncological appropriateness, has allowed the transfer of the many advantages of mini-invasiveness to the treatment of gastro-intestinal cancer. Surgeons around the world are gaining experience with advanced laparoscopic and robotic skills, new innovations and techniques in minimally invasive pancreatic surgery will evolve and this kind of approach will be considered a common surgical technique.

This review clearly demonstrates the importance of a scientifically based standardization of oncologic gastrointestinal surgery and the need of highly specialized and high-volume centers offering the appropriate surgical approach through the development of newly designed operative techniques and the introduction of better technological devices for laparoscopic surgery together with the undoubted improvement of surgical skills.

## Data Availability

Not applicable.
